# Experiences of antenatal care among pregnant adolescents at Kanyama and Matero clinics in Lusaka district, Zambia

**DOI:** 10.1186/s12978-018-0565-9

**Published:** 2018-07-09

**Authors:** Bwalya C. Bwalya, Doreen Sitali, Kumar Sridutt Baboo, Joseph M. Zulu

**Affiliations:** 1 Ministry of Education, Matero Girls Secondary School, P.O Box 32979, Lusaka, Zambia; 2 Departments of Public Health, School of Medicine, University of Zambia, P.O. Box 50110, Lusaka, Zambia

**Keywords:** Pregnant adolescents, Antenatal care, Experiences

## Abstract

**Background:**

Adolescent pregnancy is among the many public health concerns not only in Zambia but also in other parts of the world. Exploring pregnant adolescents’ experiences of antenatal care may help to identify specific problems and the contextual relevant strategies to improve the access to antenatal care. The purpose of this study was to explore and describe the lived experiences of antenatal care among pregnant adolescents aged between 12 to 19 years old at Kanyama and Matero Referral Clinics in Lusaka district of Zambia.

**Methods:**

This was a qualitative study which used in-depth interviews to collect data. Maximum variation sampling technique was used to select 12 pregnant adolescents of 12 to 19 years age range that attended antenatal care. Data were analysed thematically with the help of Nvivo software version 10.

**Results:**

The study revealed that the adolescents experienced positive and negative antenatal care. While there were some reported cases of caring and friendly health care providers and older pregnant women, there were also reported cases of poor attitudes and behaviours by the older pregnant women and health care providers towards the adolescents. In addition, other issues that were reported by the adolescents were the opening hours for the health facilities which was not favourable to all adolescents and the lack of specific spaces for adolescents as well as inadequate privacy and confidentiality. Some solutions were suggested to overcome some of the problems such as reducing the waiting hours or time for consultations at the clinic and to have specific rooms or spaces for pregnant adolescents at the clinic.

**Conclusion:**

Appropriate interventions targeting pregnant adolescents with emphasis on making antenatal care services more adolescent friendly may improve the quality of and accessibility of antenatal services. The adolescent friendly antenatal services should integrate health promotion activities aimed at sensitising elderly women within the health facilities on the importance of supporting pregnant adolescents.

## Plain English summary

There are several adolescent pregnancies in Zambia and other low and middle income countries. These adolescent pregnancies pose a serious health risk to the adolescents. Meanwhile, many of these adolescents have difficulties to access antenatal care services and comprehensive strategies to address these challenges are lacking.

Using interviews with 12 adolescent mothers, the study explored the challenges and positive issues that adolescents aged between 12 to 19 years experience when accessing antenatal care at Kanyama and Matero Referral Clinics in Lusaka district of Zambia. Also, strategies to address these challenges were documented.

Adolescents reported that the barriers to accessing health services included poor attitudes and behaviours of some older pregnant women and health care providers towards the adolescents, the opening hours for health facilities being unfavourable to most adolescents, lack of specific rooms or spaces for adolescents as well as inadequate privacy and confidentiality. In order to improve the access to the services, adolescents recommended that it was important to reduce the waiting time before consultations at the clinic and to have specific rooms or spaces for pregnant adolescents.

In conclusion, to effectively improve the access to antenatal services by pregnant adolescents, there is need for increased sensitisation of adult women and health workers on the importance of supporting adolescents during antenatal care.

## Background

Adolescent pregnancy is one of the sexual and reproductive health (SRH) problems that several low and middle income countries (LMICs) [1] experience. It accounts for about 11% of all births worldwide (WHO, 2014) and 95% of these births occur in LMICs [1]. In Zambia, approximately 3 in every 10 adolescents aged between 15 to 19 years have either given birth or are pregnant [2]. However, adolescent pregnancy is associated with various maternal health-related complications and deaths. For instance, it has been documented worldwide that pregnancy and childbirth related complications are among the leading causes of death for adolescent girls aged between 15 to 19 years [1, 3]. In addition, research has also shown that girls who give birth before the age of 16 years are three to four times more likely to suffer maternal death than women in their twenties [2].

Additionally, adolescents are more likely to suffer from other pregnancy related complications [2, 4] such as obstructed labour [5], toxemia, hemorrhage, hypertension and anaemia [6], low birth weight, premature delivery, preeclampsia and anaemia [4] among others. Research has also shown that most of these maternal-related complications mentioned above can be avoided through timely access to quality antenatal health care services as it presents an opportunity to prevent or manage pregnant related risks [7–12].

According to Chama [10], antenatal care provides a platform to inform and educate expectant mothers about pregnancy and the importance of seeking skilled health care during the time before and after birth. The goal of antenatal care is to provide regular checkups that would enable doctors or midwives to screen, prevent, detect and treat potential health complications that may arise in pregnant women [3, 6]. To be specific, it provides a platform to identify and manage any maternal-related complications or risk factors [3, 4] such as pregnancy induced hypertension, malaria and anaemia which put at risk the life of both the mother and unborn baby.

In addition, antenatal care offers a conducive environment to health care providers to interact with the pregnant women on issues affecting their health and that of the unborn child because health issues are discussed at length during antenatal classes. These issues include nutrition education, information about the risk of cigarette smoking, family planning, exclusive breast feeding, immunization, care of the newborn as well as the importance of rest, hygiene and safer sex [4]. In general, studies have shown that women want and expect positive outcomes from antenatal services. These positive experiences include *maintaining physical and sociocultural normality, maintaining a healthy pregnancy for mother and baby including preventing and treating risks, illness and death, having an effective transition to positive labour and birth as well as achieving positive motherhood that includes maternal self-esteem, competence and autonomy* [3, 4].

Despite the benefits of antenatal care, adolescents are less likely to use antenatal care services compared to older women [3, 11]. The reasons for low utilisation of antenatal care services among pregnant adolescents include several issues such as, health worker judgmental attitudes and lack of training in and understanding of adolescents’ reproductive needs [12]. There is also fear of humiliation and having to respond to unpleasant questions and procedures during antenatal care. Furthermore, there is lack of respect, privacy and confidentiality within the health care system [3]. Some parents and guardians for adolescent mothers maybe upset in the initial stages of the pregnancy as a result there is limited support from parents to adolescents with regard to proper guidance on attending antenatal care services. This lack of support and guidance is challenging especially in cases where adolescents depend on their parents for financial support to enable them access health care services [5, 13].

Adolescents or women friendly antenatal care services could be life saving as shown by studies that women may refuse to seek antenatal care from a health care provider who “abuses” them or one who does not treat them well, even if the health care provider is skilled in the prevention and management of pregnancy complications [6, 13].

Several maternal health-related studies have been conducted in Zambia [7, 9]. However, there is limited information specifically on the experiences of antenatal care utilization among pregnant adolescents. Therefore, a comprehensive understanding of lived experiences of pregnant adolescents with regard to the antenatal clinic environment will add value to the existing body of knowledge on issues surrounding maternal and child health in Zambia.

The specific objectives of this study were: (i) to describe the experiences of pregnant adolescents with the healthcare providers at the antenatal care clinic; (ii) to describe the experiences of pregnant adolescents with older pregnant women within the antenatal care clinic; and (iii) to describe the experiences of pregnant adolescents with education provided at as part of antenatal care.

## Methods

### Study design

This study was a qualitative phenomenological study. “A phenomenological study focuses on describing what all participants have in common as they experience a phenomenon” [14]. Phenomenology provides a deep understanding of a phenomenon as experienced by several individuals. Data is collected from people who have experienced the phenomenon and develops a composite description of the experience for all the individuals. In line with the phenomenological design, this study focused on describing “what” the (adolescents) experienced and “how” they experienced it [14], as opposed to proving and disapproving a hypothesis or predicting why particular adolescents had different experiences with the health care providers or with the older pregnant women in the clinics.

This design and approach allowed the researchers to explore and develop an in-depth understanding of the experiences and perceptions of pregnant adolescents attending antenatal clinics at Kanyama and Matero Referral Clinics. In particular, the design and approach enabled the researchers to uncover details about the experiences of pregnant adolescents within the antenatal clinic environment.

### Study area

The study was conducted at Kanyama and Matero Referral Clinics in Lusaka district, the Capital city of Zambia. These two clinics offer first level health care services to the surrounding communities and are both located in what are considered densely populated areas. Purposive sampling technique was used to select these two clinics based on the availability of antennal care services.

### Study sample

The research participants for this study were 12 pregnant adolescents of between 15 and 19 years old who had attended two or more antenatal care visits at their respective clinics in the two research sites. The research participants were purposively selected to take part in the study from Kanyama and Matero Referral Clinics. All the names that met the inclusion criteria were identified from the antenatal care records at the clinics. Eligible pregnant adolescents were those who (a) were antenatal care attendees at the two respective research sites; (b) were between 12 to 19 years old; and (c) those who voluntarily gave consent to participate in the study. Participants were then informed about the nature of the study during the process of recruitment. For those adolescents that were below 17 years old, their parents or guardians were then contacted for parental consent.

Maximum variation sampling frame was used as a guide to determine the sample size for the study. This type of purposive sampling was applied in order to capture maximum variation in terms of age. Eight age categories of pregnant adolescents were identified (from 12 to 19 years old). From each of the eight categories, two participants, one per site, were sampled, thus bringing the total of the initial target sample size to 16 pregnant adolescents. However, only 12 participants were interviewed (7 from Matero Referral Clinic. and 5 Kanyama Referral Clinic). This was due to the fact that no new information came out from the interviews. In addition, participants from the category of 12 to 14 years old were unavailable at the time of conducting the study.

### Data collection

Data were collected using semi-structured interviews. The interviews were conducted within the two clinic premises. A quiet and private room was used for the interviews. On average, the interviews lasted 30 minutes. All the interviews were audio recorded with permission from the participants.

### Data analysis

The audio recorded interviews were transcribed and imported into Nvivo software version 10. The software was used to facilitate data management and analysis of emerging themes. Thematic analysis was used to analyse interview transcripts. Several broad themes were predetermined based on the questions from the interview guide on the experiences of antenatal care among pregnant adolescents. Additional themes were generated from the ideas that were coming out from the responses of participants as we read through the transcripts.

### Ethical consideration

Ethical approval to conduct the study was granted by the University of Zambia Biomedical Research Ethical Committee (UNZABREC) at the University of Zambia. Approval to conduct the study at the two health facilities was also granted by the two sisters-in-charge. Voluntary written informed consent, assent and parental or guardian consent were also obtained from all the research participants.

## Results

A total of 12 pregnant adolescents aged between 15 to 19 years participated in the study. The participants were receiving antenatal care services at the time of the interviews. Direct quotations from the raw data were provided to support the description of the experiences and the findings of the study. The true identities of participants were replaced with pseudonyms (the first number represented the age of the participant and the last number on the left represented the I.D number for the participant whereas letters represented the study site: K for Kanyama Referral Clinic and M for Matero referral clinic). The results were organised as follows: Experiences with service providers;

(i) Experiences with other service users;
(ii) Experiences of antenatal education; and
(iii) Health facility related challenges.

### Experiences with health service providers

There were variations regarding the experiences of pregnant adolescents with health care providers at the clinics. While some adolescents had positive experiences, others had negative experiences. Below are the detailed explanations of their experiences with the health care providers.

#### Good reception by service providers, a motivating factor to accessing services

Most study participants from both Kanyama and Matero Referral Clinics revealed that their relationship with the health care service providers was good. This good relationship was attributed to the good reception and the general care that adolescents received from health providers. An 18 year-old participant from Kanyama Clinic explained this good relationship as follows:

“*I have a good relationship with the nurses. They welcomed me very well the first-time l came here with my husband and they are very educative” 18-K10*

### Negative health worker attitudes discouraging health service utilisation

Although some pregnant adolescents had good experiences with the health care providers, other participants encountered negative attitudes and behaviours from them during the antenatal care service. Some adolescents complained that the nurses were sometimes rude in the way they communicated with them. One participant reported that one nurse was rude in the way she spoke to her when she stated that:

*“The first time the nurse was rude because I did’nt understand her instructions clearly” 15-K8*

Another research participant echoed a similar negative experience with regards to the attitudes and behaviour of health care providers at Matero Clinic. The doctor shouted at her for not waiting for two years before conceiving the second time. Below is how the adolescent explained her situation:

“*I had a pregnancy last year that ended up in an operation but I lost the baby. When I came back for antenatal this year I requested for a referral to go to another hospital from the in charge.. Instead, she started scolding at me for having not waited for 2 years before getting pregnant again… I was hurt with the words she used,” 19-M12.*

The participants also felt that they were being discriminated against by the health care providers because of their marital status. The reasons cited for this was that health workers gave first priority to those that went with their partners for antenatal care. They complained that the nurses first attended to the couples before they attended to the adolescents despite reporting to the clinic much earlier than the said couples:

*“I don’t like the way this clinic operates because they give first priority to those women who come with their husbands… even when they come late for antenatal care” 16-K11.*

### Experiences with other service users

The participants narrated different experiences with older pregnant women with whom they attended the same antenatal classes at their respective clinics. Some adolescents had positive experiences while others experienced stigma and unfriendly interactions with the older women.

#### The integration of adolescents and older women facilitates exchange of experiences

At both Kanyama and Matero Referral Clinics, there is no separation between adolescents and the older women in antenatal classes. The participants in this study expressed different views on how they related with the older women during antenatal care. Generally, most of the research participants reported that they had healthy relationships with older expectant mothers. Some adolescents reported that the older women provided them with advice on how to take care of their pregnancy and that they got along very well during antenatal care as one adolescent said:

*“l ask them pregnancy related questions… They teach me on how to take care of my pregnancy such as, what to eat, not to sleep too much and carry heavy things and preparations for my delivery….. They teach me things l don’t know” 19-M2*.

However, a few pregnant adolescents reported that during their interaction with the older women, they encountered negative experiences. Older women were perceived to be unfriendly or uninterested in the adolescents when they did not initiate or engage themselves in any conversations with the adolescents. Such encounters with the older women at the clinics made the adolescents feel out of place, uncomfortable, ashamed and discouraged to attend antenatal care services:

*“Others don’t really want to be talked to but others are very interesting and welcoming…. l feel lonely because some women are not friendly. They spend most of their time on the phone” 19-M9.*

Experiences of stigma were also indicated as additional challenges faced by the adolescents at the clinic. Some adolescents complained of lack of respect by the older women. These experiences of lack of respect often arose through their contact with the older women who came for the same services. According to the adolescents, this lack of respect made them feel ashamed and uncomfortable thereby triggering stigmatization:

“*I overheard some pregnant women talking in a mocking manner when I went to call my husband. This made me to feel uncomfortable and ashamed of myself. They were even laughing at me” 15-K8.*

### Experiences of antenatal education

There were different views on the usefulness of antenatal education by the participants. Overall, the participants highlighted their positive learning outcomes of antenatal education. These positive outcomes mainly focused on prevention, treatment as well as management of diseases.

#### Antenatal education very beneficial

When the issue of what was learned at antenatal was explored, the majority of the participants said that they learnt on how to take good care of themselves during pregnancy. They had learnt about HIV prevention and management, and in particular how the risk of mother to child transmission could be reduced.

*“They teach us about HIV...how one can get HIV… and how to take care of ourselves when you are pregnant. I share this information with others what l learn here” 16-M1*

Several other learning outcomes from the interviews were identified and these revolved around facts concerning prevention of malaria, what constitutes a healthy diet for both the mother and the unborn child, the effects of drinking or smoking on the baby and the mother and preparations for delivery.

*“I learnt that a pregnant woman should not smoke or drink alcohol” 17-K6*

#### Operating hours for antenatal care a challenge for some adolescents

Several concerns regarding the operating hours of the antenatal clinic were expressed by the study participants. Long waiting hours in the queue for consultation as well as long periods of time spent at the clinic were among the many concerns raised by the adolescents.

There are specific services that are routinely provided at Kanyama and Matero Referral Antenatal Clinics such as weight measurement, physical assessment of pregnancy, laboratory tests and immunizations. The standard operating hours for antenatal care for both Kanyama and Matero Referral Clinics is between 08:00 to 13:00 h. When asked what time, they arrived at the clinic for antenatal care, the participants gave different responses. The majority of the participants arrive at the clinic before 07:30 h, while others come at 08:00 h.

*“I have to come as early as 06:00 hours” 17-K6*

Pregnant adolescents also had different opinions regarding their preferred time frame to spend at the antenatal care. Some participants suggested 08:00 to 12:00 h as the most convenient time to spend at the clinic so that they could have enough time to do other things at home.

*“08:30–12:00 or14:00 is ok with me so that l can go home and sleep because the fansidar we take make us drowsy” 19-M2*

#### Too early at the health facility and yet being attended to late

Some participants complained that sometimes they could wait for about 2 to 3 h during the official antenatal clinic operating hours before they could be attended to by health workers. This was attributed to health care providers performing other tasks before attending to them.

*“I wait for long hours before they begin antenatal class…. about 2 to 3 hours” 15-K8*

Other research participants also complained about finishing with the clinic as late as 16:00 h in the afternoon despite them going to the clinic very early in the morning. As a result of this they felt hungry.

*“I don’t like it. I come around 06:00 h I sometimes have to go home at 16:00 h and I usually get very hungry” 17-K7*

### Health facility related challenges

#### Lack of adolescent specific spaces

Furthermore, all the study participants reported that the clinics did not provide separate space for antenatal care specifically for adolescents nor did they provide a waiting room for adolescents only. This meant that the pregnant adolescents utilised the same antenatal care services as the older women.

*“No, the clinic has no separate space to provide services for adolescents” 18-M5*

Some participants felt uncomfortable to share the same spaces with the older women.. . Adolescents also viewed this practice as a sign of lack of respect to the older women due to lack of privacy during the physical assessments of pregnancy.

*“No there is no separate room for adolescents that come for antenatal care. We are mixed together with the older pregnant women….l don’t like this arrangement because people lose respect. There are times were you see an older woman undressing when the nurse is conducting physical assessments of pregnancies or during delivery in the labour ward… …..Such is not a good thing to experience because she is like my mother”19-M2*

*“l feel shy when am with older women”18-K10*

The pregnant adolescents were put in the same antenatal class as the older pregnant women. Other participants reported that the older women had the tendency of dominating the class discussions and would pass derogatory comments against the adolescents. Some participants did not feel free to participate in the lessons due to the sensitive nature of certain sexual topics. Discussing sexual issues in the presence of adults made the adolescents not to ask questions because they felt shy as attested to by a participant when she said:

*“There are always embarrassing topics especially when you come with your partner… about private parts. I do not ask questions during class because I feel shy. I would only ask questions privately” 19-M9*

Some participants preferred to attend antenatal classes just for adolescents. Such classes would enable them to freely express themselves and to feel comfortable because they would be with their age mates. Having a specific class for adolescents would enable them to learn more on pregnancy and the related outcomes. To support this assertion, a participant stated that:

*“l would like to have a separate class specifically for adolescents because such an arrangement can make me feel free. l will be with my age mates…I am not free to ask questions because l feel shy” 17-K7*

#### Inadequate privacy and confidentiality

Issues of privacy and confidentiality were also brought out by the adolescents. Some participants reported that the counseling and screening sessions were conducted in a private and confidential environment. They felt confident that the sensitive information that they discussed with the health care providers was not overheard or retold to other persons as indicated in the quote hereunder that:

*“Yes there is privacy when we come for HIV counseling and testing. No one hears what we discuss during pre-testing and post-testing”19-M3*

Other participants however reported that the counselling and examination rooms at the clinic did not guarantee privacy and confidentiality. One adolescent for example, reported that one could hear from outside what the patient and the counsellor were talking about. Lack of privacy was also raised by another participant when she reported that nurses kept on interrupting the counselling session when she said:

*“The rooms are not very private because the people outside are able to hear what is being discussed between the counsellor and the patient”18-M4*

#### Overcrowded environment

The other concern was overcrowding due to antenatal classes and children’s under five clinics being conducted in the same room and at the same time. Some adolescents reported that they did not pay attention to what was being discussed in the antenatal classes because of the noise that came from the mothers and children that were being attended to as well. It was alleged that:

*“We are in the same room with new recruits, the old ones and those that come for under five. This is not good because there is a lot of noise and l can’t really hear what we are learning…. So they need to find another place so that we learn more. It’s crowded” 19-M9*

## Discussion

The findings demonstrated that pregnant adolescents had both positive and negative experiences of antenatal care services. Some adolescents expressed appreciation for the healthy relationship they had with the health care providers. A study conducted in the community drop-in program for young women who were pregnant or had an infant below 6 months of age in Canada showed that providing care to adolescents in a friendly manner is important to them [15].

However, other participants felt that they were being discriminated against by health care providers especially when they gave priority to the older couples despite their reporting late. They also described some health care service providers as being rude and disrespectful in the way they communicated with the adolescents. Similar findings were reported in a study done by Duggen and Adejumo [16]. The adolescents, who were recruited from antenatal and postnatal services in South Africa, wanted health care services to be carried out in the same manner as it would be for the adult women.

Ways in which pregnant adolescents interacted with the older pregnant women were revealed. Older women were perceived to be unfriendly when they did not initiate or engage themselves in any conversations with the adolescents. Such encounters with the older women at the clinic made the adolescents feel out of place, uncomfortable, felt a sense of shame and discouraged to even to attend subsequent antenatal care visits. In such an environment, individuals may experience loneliness when they feel that their relationships with others are less satisfying than they would like them to be [6]. Similar findings were also documented [5, 17]. These authors argued that adolescents are fully aware that society tended to judge and discriminate against women who appeared to violate the perceived social or moral conventions such as having sex at a tender age. These social and moral conventions often stemmed from various societal, cultural and religious belief systems that are deeply embedded in the communities. It is therefore recommended that efforts should be made to make pregnant adolescents comfortable within the health care system that is aimed at ensuring that they continued to access appropriate care services and to minimise potential for feeling socially isolated and devalued [16].

Although there is limited information specifically on experiences of antenatal care among adolescents in Zambia, a few studies conducted in antenatal care clinics elsewhere reported findings that are consistent with those in this study [6, 16]. For example, pregnant adolescents perceived adult-oriented antenatal care to be uncomfortable because they shared the same class with the older women. Our findings suggest that pregnant adolescents were unable to freely participate in group antenatal class discussions due to the sensitive nature of certain sexual topics in the midst of adult women and their partners. Consequently, they experienced fear, embarrassment and uneasiness. According to Svanemyr et al. [18], adolescent girls are less likely than the older women to ask, obtain information, discuss and express their worries about sexual and reproductive health issues due to the culture of silence which is deeply embedded in the social norms and taboos related to sexuality in families or the communities. This is particularly the case when it comes to communication with adults either at the clinic or at home.

Some participants complained that they received antennal care services late despite arriving early at the clinic. Other participants complained of the long waiting hours at the health facility. The adolescents made suggestions on the actual time that would best suit them to spend at the clinic. The same concerns were also expressed in other studies [5, 6, 16, 19]. Addressing such delays at the clinic may motivate adolescents to regularly access antennal care services.

### Strengths and limitations

This study added useful insight to the body of knowledge on issues surrounding adolescents’ maternal health in Zambia. However, the study experienced methodological challenges. Firstly, the study was unable to find pregnant adolescents that represented adolescents from 12 to 14 years old. Only older adolescents (18–19 years old) were the majority participants. Therefore, this study was deprived of insights from the experiences of pregnant adolescents between the ages of 12 to 14 years as they may have had different experiences. Last but not the least, these experiences of antenatal care services among pregnant adolescents cannot be generalised because there are only limited to two health facilities. Therefore, an additional study should be conducted in other clinics outside Lusaka district to explore their experiences of antenatal care services provided to pregnant adolescents.

## Conclusion

This study showed that adolescents had both positive and negative experiences with antenatal care services at Kanyama and Matero Referral Clinics. The positive experiences were attributed to a few friendly health care providers and the older expectant mothers who attended the same antenatal care services with the adolescents. Stigma and discrimination, long waiting hours at the clinic and lack of adolescent spaces were among the cited negative experiences among the research participants. Appropriate interventions targeting developing more adolescent friendly maternal health services which should integrate provision of health education to the older women to support pregnant adolescents may result in improved quality of antenatal care and subsequently increased utilisation of the services by adolescents.

## Abbreviations

DHMT: University of Zambia and District Health Management Team
LMICs: Low and Middle Income Countries
SRH: Sexual and Reproductive Health
UNZABREC: University of Zambia Biomedical Research Ethical Committee
WHO: World Health Organisation

## References

[1] Morris JL, Rushwan H (2015). Adolescent sexual and reproductive health: the global challenges. Int J Gynecol Obstet.

[2] Zambia Adolescent Health Strategic Plan (2011-2015), Ministry of Health, Government of the Republic of Zambia.

[3] World Health Organisation. WHO recommendations on antenatal Care for a Positive Pregnancy Experience. Geneva: WHO; 2016. 28079998

[4] Downe S, Finlayson K, Tunçalp Ӧ, Metin Gülmezoglu A (2016). What matters to women: a systematic scoping review to identify the processes and outcomes of antenatal care provision that are important to healthy pregnant women. BJOG Int J Obstet Gynaecol.

[5] Bhandari SD, Joshi S (2017). Perception and perceived experiences about prevention and consequences of teenage pregnancy and childbirth among teenage mothers: a qualitative study. Journal of Advanced Academic Research.

[6] James S, Rall N, Strumpher J (2012). Perceptions of pregnant teenagers with regard to the antenatal care clinic environment. Curationis.

[7] Banda I, Michelo C, Hazemba A (2012). Factors associated with late antenatal care attendance in selected rural and urban communities of the Copperbelt Province of Zambia. Medical Journal of Zambia.

[8] Fernandes RFM, Meincke SMK, Thumé E, Soares MC, Collet N, Carraro TE (2015). Characteristics of antenatal Care for Adolescents from state capitals in southern and North-Eastern Brazil. Texto and Contexto-Enfermagem.

[9] Kyei NN, Campbell OM, Gabrysch S (2012). The influence of distance and level of service provision on antenatal care use in rural Zambia. PLoS One.

[10] Chama-Chiliba CM, Koch SF (2015). Utilisation of focused antenatal Care in Zambia: examining Iindividual- and community-level factors using a multilevel analysis. Health Policy Plan.

[11] Dairo M, Owoyokun K. Factors affecting the utilisation of antenatal Care Services in Ibadan, Nigeria. Benin Journal of Postgraduate Medicine. 2010;12(1). https://www.ajol.info/index.php/bjpm/article/view/63387. Accessed 6 July 2018.

[12] Atuyambe L, Mirembe F, Tumwesigye NM, Annika J, Kirumira EK, Faxelid E (2008). Adolescent and adult first time Mothers' health seeking practices during pregnancy and early motherhood in Wakiso district, Central Uganda. Reprod Health.

[13] Gyesaw NYK, Ankomah A (2013). Experiences of pregnancy and motherhood among teenage mothers in a suburb of Accra, Ghana: a qualitative study. International Journal of Women's Health.

[14] Lewis S (2015). Qualitative inquiry and research design: choosing among five approaches. Health Promot Pract.

[15] Peterson WE, Sword W, Charles C, Dicenso A (2007). Adolescents' perceptions of inpatient postpartum nursing care. Qual Health Res.

[16] Duggan R, Adejumo O (2012). Adolescent clients’ perceptions of maternity Care in Kwazulu-Natal, South Africa. Women and Birth.

[17] Weed K, Nicholson JS (2015). Differential social evaluation of pregnant teens, teen mothers and teen fathers by university students. International Journal of Adolescence and Youth.

[18] Svanemyr J, Amin A, Robles OJ, Greene ME (2015). Creating an enabling environment for adolescent sexual and reproductive health: a framework and promising approaches. J Adolesc Health.

[19] Michels TM (2000). "patients like us": pregnant and parenting teens view the health care system. Public Health Rep.

